# C-STICH: Cerclage Suture Type for an Insufficient Cervix and its effect on Health outcomes—a multicentre randomised controlled trial

**DOI:** 10.1186/s13063-021-05629-3

**Published:** 2021-09-28

**Authors:** Fidan Israfil-Bayli, Victoria Hodgetts Morton, Catherine A. Hewitt, Andrew K. Ewer, Jim Gray, Jane Norman, Christoph Lees, Nigel A. B. Simpson, Andrew Shennan, Konstantinos Tryposkiadis, Max Hughes, Jane Daniels, Peter Brocklehurst, Katie Morris, Lee Middleton, Philip Toozs-Hobson

**Affiliations:** 1 Birmingham Women’s and Children’s Hospital, Birmingham, UK; 2 University of Birmingham, Birmingham, UK; 3 University of Bristol, Bristol, UK; 4 Imperial College London, London, UK; 5 University of Leeds, Leeds, UK; 6 Kings College London, London, UK; 7 University of Nottingham, Nottingham, UK; 8 Birmingham Clinical Trials Unit, Birmingham, UK

**Keywords:** Obstetrics and gynaecology, Preterm birth, Cervical cerclage

## Abstract

**Background:**

Preterm birth is associated with significant mortality and morbidity for mothers and babies. Women are identified as high risk for preterm birth based on either previous medical/pregnancy history or on ultrasound assessment of the cervix. Women identified as high risk can be offered a cervical cerclage (a purse string stitch) around the cervix (neck of the womb) to reduce the risk of preterm birth. In women who have a cervical cerclage, the procedure can be performed using either a monofilament (single-stranded) or braided (woven) suture material. Both suture materials are routinely used for cervical cerclage and there is uncertainty as to which is superior.

**Methods:**

A multicentre, open, randomised controlled superiority trial of 2050 women presenting at obstetric units, deemed to be at risk of preterm birth and already scheduled to have a cervical cerclage as part of their standard care. Inclusion criteria include singleton pregnancies and an indication for cervical cerclage for either a history of three or more previous mid-trimester losses or premature births (≤ 28 weeks), insertion of cervical sutures in previous pregnancies, a history of mid trimester loss or premature birth with a (current) shortened (≤ 25 mm) cervix, or women whom clinicians deem to be at risk of preterm birth either by history or the results of an ultrasound scan. Exclusion criteria include women who have taken part in C-STICH previously, are aged less than 18 years old at the time of presentation, require a rescue cerclage, and are unwilling or unable to give informed consent and in whom a cerclage will be placed by any route other than vaginally (e.g. via an abdominal route). Following informed consent, women are randomised on a 1:1 basis to either monofilament or braided suture, by minimisation. The primary outcome is pregnancy loss (miscarriage and perinatal mortality, including any stillbirth or neonatal death in the first week of life), and secondary outcomes include the core outcome set for preterm birth trials.

**Discussion:**

Optimising established interventions to prevent preterm birth is important in reducing perinatal mortality rates.

**Trial registration:**

ISRCTN 15373349. Registered before recruitment on 03 December 2014 prior to first recruit.

## Administrative information

Note: the numbers in curly brackets in this protocol refer to SPIRIT checklist item numbers. The order of the items has been modified to group similar items (see http://www.equator-network.org/reporting-guidelines/spirit-2013-statement-defining-standard-protocol-items-for-clinical-trials/).

**Table:** 

Title {1}	C-STICH: Cerclage Suture Type for an Insufficient Cervix and its effect on Health outcomes. A multicentre randomised controlled trial.
Trial registration {2a and 2b}.	ISRCTN15373349 10.1186/ISRCTN15373349 First registered 03/12/2014 prior to recruitment opening
Protocol version {3}	Protocol version 9.0, 25^th^ March 2020
Funding {4}	NIHR HTA funded trial, trial reference number 13/04/107
Author details {5a}	FIB developed and wrote the initial protocol, with input from the collaborative team listed as authors. VHM has worked as the clinical research fellow since prior to recruitment and developed the trial design from protocol version 1 to 9 and prepared the manuscript for submission. PTH is the Chief investigator of the trial and has provided oversight to conduct and protocol development. CH and LM are the trial statisticians and have had input to trial design and outcome measures. JD and PTB have provided CTU input. JG has advised regarding the microbiology component, AKE has advised and supported the collection of the neonatal outcomes. JN, NS, AS, AKE, CL, KM are co-applicants on the trial and have supported development of the trial design and recruitment.
Name and contact information for the trial sponsor {5b}	Birmingham Women’s and Children’s NHS Foundation Trust (BWCNFT) Sponsor contact details: Sarah Hatfield
Role of sponsor {5c}	The sponsor is the grant holder for this study. The sponsor has not been involved in the design of the study, analysis and interpretation of data. A sponsor representative is a member of the Trial Management Group and is involved in discussion and decision making regarding the day to day study management, study conduct, report writing and proposed publications.

## Introduction

### Background and rationale {6a}

Preterm birth (PTB) is one of the major global challenges in obstetrics and neonatology. An early birth puts survivors at risk of serious long-term disabilities and these outcomes pose a significant burden on parents as well as having economic implications on health services. Second trimester miscarriage and preterm birth form a continuum with the underlying cause often multifactorial and complex [1]. Cervical weakness is one cause of late miscarriage and preterm birth; this can be treated by a cervical cerclage.

A cervical cerclage can be performed either with a monofilament or braided suture material; both sutures are routinely used by clinicians [2]. A monofilament suture may be superior at preventing miscarriage, stillbirth and neonatal death through the prevention of early delivery [3].

## Objectives {7}

### Primary objective

- To examine the effect of using monofilament suture material compared with braided suture material on pregnancy loss rate in women deemed to be at risk of an insufficient cervix and treated with a cervical cerclage.

### Secondary objectives

- To assess the effect of suture material on other pregnancy and neonatal outcomes.
- To explore the variation in treatment effect between McDonald’s and Shirodkar’s cerclage, especially with reference to bladder dissection.
- To explore the variation in treatment effect between the different indications for cerclage.

## Trial design {8}

C-STICH is a multi-centre, parallel-group randomised controlled superiority trial, open to all maternity sites across the UK. Women are potentially eligible for C-STICH if they are attending antenatal clinics or admitted to obstetric wards in which the reviewing clinician believes that the placement of a cervical cerclage is the most appropriate treatment to prevent a miscarriage or preterm birth. Women will be invited to take part in the C-STICH trial after the decision is made to perform a cervical cerclage procedure providing that they meet the eligibility criteria detailed below.

## Methods: participants, interventions, and outcomes

### Study setting {9}

The trial is being open to all NHS maternity sites across the UK. A full list of trial sites can be obtained by contacting the Birmingham Clinical Trials Unit.

### Eligibility criteria {10}

Women are eligible if they are undergoing a cervical cerclage as part of their standard care.

#### Inclusion

- Singleton pregnancy
- Indication for cervical cerclage for either:
  ▪ A history of three or more previous midterm losses or premature births (≤ 28 weeks), OR
  ▪ Insertion of cervical sutures in previous pregnancies, OR
  ▪ A history of mid-trimester loss or premature birth with a (current) shortened (≤ 25 mm) cervix, OR
  ▪ Women whom clinicians deem to be at risk of preterm birth either by history or the results of an ultrasound scan

#### Exclusion

- Women who have taken part in C-STICH previously
- Women aged less than 18 years old at the time of presentation
- Those with a multiple pregnancy
- Those requiring a rescue cerclage*
- Women who are unwilling or unable to give informed consent
- Those in whom a cerclage will be placed by any route other than vaginally (e.g. via an abdominal route)
- Immediate need for insertion of a suture**
- Women who have membranes that have ruptured or are surfacing***

*For study purposes, rescue cerclage is defined as emergency cerclage where sutures are inserted in women who have had their preterm labours (e.g. uterine contractions, progressive cervical dilatation, bulging membranes) sufficiently halted by tocolysis or other means between 15 and 28 weeks.

**Immediate need for insertion of a suture should not be delayed by the trial (thus, if giving information about the trial and waiting for the participant to decide upon whether or not she wants to participate will delay the insertion of an urgently needed suture, then treatment should go ahead and the woman should be excluded from the trial).

***Women with membranes that are ruptured or bulging through the external os are considered to be undergoing a rescue cerclage and be excluded from trial participation.

If a woman is screened but is not eligible for the trial, either due to a preference for a particular suture type, a contraindication to the trial, a pathological reason, or consent for randomisation is not given, an anonymous record of the case should be kept in the screening log. The screening log will collect initials, age group, ethnic group, and the reason each patient is not eligible to participate in the trial. Women who consent and are subsequently found to be ineligible should also be noted. The screening log should be kept in the site file and a copy sent to the C-STICH Trial Office on a monthly basis. Members of the trial co-ordination team will be unable to identify women based on the information provided. This screening log information will inform updates to the funder regarding recruitment targets for C-STICH Trial.

The clinician performing the cervical cerclage should be competent to perform the procedure and should routinely perform cervical cerclages as part of their standard practice.

### Who will take informed consent? {26a}

Potentially eligible women will be identified in antenatal clinics, outpatient departments, and obstetric wards and invited to join the trial after a decision is made to undertake a cervical cerclage.

#### Approaching potential participants for consent

Potential participants will only be approached by suitably qualified and experienced personnel who are recorded on the delegation log.

#### Obtaining consent

All women who are referred to secondary care for cervical cerclage should be considered potentially eligible and should be screened prior to their antenatal appointment by the designated C-STICH staff member in each centre. The obstetrician who will be providing the woman’s clinical care and performing the procedure will discuss preventative options and establish eligibility based on history and preferences.

The indication for the cerclage should be discussed before the trial is introduced. The trial should then be introduced by a suitable qualified staff member who is delegated to discuss the trial and recorded on the delegation log to do so. A Patient Information Sheet (PIS) should be given to the women. Consent should be informed and voluntary with time for questions and reflection. Adequate time should be allowed for consideration of participation in the trial before the cerclage procedure is undertaken. There is no pre-agreed specified time to consent.

Consent to participate in C-STICH will be sought by appropriately qualified staff who are both GCP trained and designated by the Principal Investigator to conduct informed consent procedures. Women will be asked to confirm their consent to participate in the C-STICH trial by initialling the appropriate boxes on the consent form and signing the form in the presence of the person taking consent.

A copy should be given to the women: one to be kept in the patient notes, the original should be kept in the local site file, and one copy sent to the C-STICH Trial Office at the University of Birmingham Clinical Trials Unit. The date the informed consent discussion took place should be recorded in the participant’s medical notes and the version number of the PIS provided to the participant.

Following consent, the woman’s GP should be notified using the study template letter.

### Additional consent provisions for collection and use of participant data and biological specimens {26b}

Routine microbiology samples, i.e. a high vaginal swab and the removed suture thread, will be collected for microbiology analysis. Consent for sample collection is included in the trial consent. Samples will be delivered to each participating hospital’s local diagnostic microbiology laboratory where they will be processed in accordance with local standard operating procedures and details of microorganisms cultured will be recorded. In laboratories which do not routinely process suture materials, they will be asked to place the suture in 3–5 ml of sterile 0.9% saline. After sonication or vigorous vortexing, 0.1-ml volumes should be cultured on appropriate agar plates for culture of aerobic and anaerobic bacteria and fungi.

### Interventions

#### Explanation for the choice of comparators {6b}

Following randomisation, a cerclage will be placed using either a monofilament or braided suture. Both types of suture used in C-STICH are standard surgical materials already in use. The suture material used must be CE marked for this purpose. The MHRA have confirmed that C-STICH is not classed as a device trial.

If randomised to the comparator braided group, the cervical cerclage should be performed using any appropriate braided suture type. There are different brands of each suture material and whilst the trial does not specify which brand a surgeon should use, the use of Mersilene® is encouraged in the braided group in order to standardise the suture types as far as possible. Mersilene® is a nonabsorbable, braided, sterile surgical suture composed of poly-ethylene terephthalate.

#### Intervention description {11a}

If randomsied to the intervention group, the cervical cerclage should be performed using a monofilament suture type. To standardise the intervention group as far as possible, Ethilon, a nonabsorbable, monofilament, sterile surgical suture composed of the long-chain aliphatic polymers Nylon 6 and Nylon 6,6 is recommended.

#### Criteria for discontinuing or modifying allocated interventions {11b}

Once a cervical cerclage is placed using the allocated suture thread, this cannot be modified or discontinued.

#### Strategies to improve adherence to interventions {11c}

To ensure adherence to the allocated suture material an email will be sent to all trial staff at the randomising centre detailing the allocation. We also recommend the allocated suture material being kept with the maternal medical records following allocation before insertion.

#### Relevant concomitant care permitted or prohibited during the trial {11d}

##### Cerclage technique

The technique of suture insertion (i.e. with or without bladder dissection) will be at the surgeon’s discretion and may include an additional occlusion stitch to close the external os. Should an additional occlusion stitch be used, we request that the same suture type as the randomised allocation be used. Technique of suture insertion will be included in the minimisation algorithm to ensure balance in treatment allocation for those who do and do not have a bladder dissection.

##### Other treatments

Apart from the suture material used during the cervical cerclage, all other aspects of the surgical procedure are at the discretion of the surgeon performing the procedure. Ongoing preterm birth prevention management, e.g. progesterone, antibiotics, and tocolytics, will be at the discretion of the clinician providing care and can all be used as required. Intention to commence progesterone will be included in the minimisation algorithm.

In rare instances, a second cervical cerclage may be required later in the pregnancy. In this case, the participant cannot be randomised a second time but should ideally be treated with the same suture material used in the initial cerclage.

The pregnancy should be managed as per current usual practice for women with a cervical cerclage, with no other special treatments, no special investigations, and no extra follow-up visits outside those required clinically are required for trial participation.

### Provisions for post-trial care {30}

There is no anticipated additional harm for women participating in C-STICH; both suture threads are routinely used in clinical practice.

### Outcomes {12}

C-STICH collects all the outcomes detailed in the core outcome set for preterm birth [4]. The primary outcome measure is pregnancy loss (miscarriage and perinatal mortality, including any stillbirth or neonatal death in the first week of life). A key secondary outcome in the C-STICH trial is time from conception to pregnancy end (any reason). Other maternal and foetal secondary outcomes include:

#### Maternal

- Miscarriage and previable neonatal death (defined as delivery <24 weeks)
- Stillbirth (defined as intrauterine death ≥24 weeks)
- Gestation at delivery (in live births ≥ 24 weeks)
- Gestational age at delivery (in live births ≥ 24 weeks)
- Gestational age <28/<32/<37 weeks at delivery (in live births ≥24 weeks)
- Time from conception to onset of spontaneous vaginal delivery (in live births ≥24 weeks)
- Sepsis (at any time in pregnancy and until 7 days postnatal)
- Preterm pre-labour rupture of membranes (PPROM)
- Mode of initiation of labour (spontaneous or induced)
- Mode of delivery (vaginal, operative vaginal or caesarean)
- Cerclage placement complications (cervical laceration/bleeding from cervix/ruptured membranes/bladder injury)
- Cerclage removal complications (cervical tears/need for anaesthetic/difficult to remove)
- Other maternal complications: vaginal bleeding/steroid use/chorioamnionitis/maternal pyrexia of 38°C (intrapartum/postnatal)/ admission to HDU or ITU (pre/post-delivery)
- Serious adverse events

#### Neonatal

- Early neonatal death (defined as a death within 7 days after delivery)
- Late neonatal death (defined as a death beyond 7 days and before 28 days after delivery)
- Birth weight adjusted for gestational age and sex (in live births ≥24 weeks)
- Small for gestational age and sex (<10th centile, in live births ≥24 weeks)
- Resuscitation at birth/additional care required (SCBU/NICU/HDU/transitional)/length of stay in additional care (in live births ≥24 weeks)
- Antibiotics within 72 h/sepsis (clinically diagnosed/proven) (in live births ≥24 weeks)
- Early neurodevelopmental morbidity (severe abnormality on cranial ultrasound scan) (in live births ≥24 weeks)
- Respiratory support (ventilation/CPAP)/days on respiratory support (in live births ≥24 weeks)
- Supplementary oxygen requirements at 36 weeks post menstrual age(in live pre-term births ≥24 weeks, <36 weeks)
- Necrotising enterocolitis (Bell’s stage 2 or 3) (live pre-term births ≥24 weeks, <37 weeks)
- Retinopathy of prematurity requiring laser treatment/disabilities/congenital abnormalities (live pre-term births ≥24 weeks, <37 weeks)
- Serious adverse events

### Participant timeline {13}

The trial is pragmatic in nature and requires no additional visits or assessments.

#### Randomisation

At the time of consent, a full gynaecological and obstetric clinical history of the woman will be taken. These details should be recorded on the Randomisation Notepad (CRF 1) prior to randomisation as these will be needed for minimisation. This form also contains a checklist for eligibility into the trial and is completed prior to randomisation.

#### Cerclage Placement

The local approved designated C-STICH staff member should report details of the cerclage placement procedure using the Cerclage Placement Form (CRF 2), providing information to include the taking of a microbiological swab, use of and type of antiseptics, technique used, the suture thread used, the number of “bites”, the position of any knots, and use of any tocolytic agent.

#### Cerclage Removal

Please use the Cerclage Removal form (CRF 3) to record details of the removal of sutures. The Cerclage Removal form has been designed to also collect information about further cerclages that have been placed since placement of the initial cerclage.

#### Microbiology Assessment

The Microbiology Assessment form (CRF 4) should be used to record outcomes of microbiology investigations done on any swabs and suture thread/s taken. Please use a separate case report form to record microbiology outcomes for either the high vaginal swab or the removed suture/s.

#### Pregnancy Outcome and Maternity Outcome

The Pregnancy Outcome and Maternity Outcome forms (CRF 5, PART A and PART B) should be used to record the primary outcome and secondary maternal outcomes at the conclusion of the pregnancy.

#### Baby Outcome

Please use the Baby Outcome form (CRF 6) to record information about the baby’s status. Only complete this form where you recorded a “live birth” on CRF 5, PART A, the Pregnancy Outcome form.

The end of neonatal follow-up will be different for neonates born preterm compared with term.

For preterm neonates (born less than 37 weeks), the end of trial follow-up will be the estimated date of delivery (as confirmed by first trimester ultrasound) or discharge from hospital whichever occurred sooner.

For term neonates (born after 37 weeks), the end of trial follow-up will be 28 days post-delivery or discharge from hospital whichever occurred sooner.

The baby outcome form should be completed at either of the above time points or at discharge from hospital whichever is soonest.

The end of trial for the *mother* will be 28 days post-delivery or death, whichever occurred sooner.

The study will be deemed complete when all participants and their neonates have reached the defined time points.

### Sample size {14}

The original sample size for C-STICH was informed by a meta-analysis of audit data, with allowance for the fact that this evidence was non-randomised. The pregnancy loss rate was 7.1% with monofilament sutures compared to 19% with braided sutures, a relative reduction of 66% (risk ratio (RR) 0.34, 95% confidence interval (CI) 0.18 to 0.63). A total sample of 326 women would be sufficient to detect a difference of this size with 90% power (*p*=0.05). However, we inflated this to a total sample target of 900 (including inflation for an attrition rate of 2.5%) which enabled us to detect a more plausible relative reduction of 41% (19% with braided to 11.2% with monofilament) with 90% power (*p*=0.05).

Given that there was uncertainty around the estimates used for the rates of pregnancy loss, it was agreed that the Data Monitoring and Ethics Committee (DMEC) would monitor the overall (pooled) event rate throughout the study to assess any deviation from the original sample size assumptions. In July 2017, the DMEC disclosed that the current estimate of the pooled event rate was lower than anticipated and may affect the trials ability to detect a difference between groups, should one exist. They advised that to maintain 90% power to detect the same relative reduction of 41% the sample size should be increased to 2050 women.

### Recruitment {15}

Recruitment will be achieved by a large network of 75 maternity sites across the UK approaching all women undergoing an elective or ultrasound indicated cervical cerclage. Eligible participants will be identified through specialist preterm birth clinics where appropriate to ensure recruitment target are met. Recruitment commenced in August 2015.

### Assignment of interventions: allocation

#### Sequence generation {16a}

Participants should be randomised just prior to the cervical cerclage procedure, to minimise the number of withdrawals and protocol violations, but allowing sufficient time for the obstetrician to prepare the sutures for the procedure. Birmingham Clinical Trials Unit provide a bespoke web-based randomisation with telephone back-up. Patients are entered and randomised into the trial by logging into secure online webpage available at www.birmingham.ac.uk/C-STICH. Each GCP-trained researcher eligible to randomise will be provided with a unique username and password. The online randomisation is available 24 h a day, 7 days a week apart from short periods of scheduled maintenance and when there are occasional network interruptions. Alternatively, investigators can make a Freephone telephone call (Tel - 0800 953 0274) to the randomisation service. This telephone randomisation service is available between 0900 and 1700 h Monday to Friday.

A Randomisation Form (CRF1) should be used to collate the necessary information prior to randomisation. All eligibility criteria and baseline data items on CRF1 will need to be answered before a trial number and allocation can be given. If an essential data item is missing, randomisation will be suspended but can be resumed once the information is available. This will be followed by a confirmatory email sent to the randomising person, local Principal Investigator, and the main research staff member within the centre.

Women will be randomised in a 1:1 ratio to either monofilament or braided suture material. A minimisation algorithm will be used to ensure balance in the treatment allocation by site, indication for cerclage (a history of ≥3 previous midterm losses or premature births (≤28 weeks)/insertion of a cervical suture in a previous pregnancy/a history of mid trimester loss or premature birth with a (current) shortened cervix (≤25mm)/women whom clinicians deem to be at risk of preterm birth either by history or the results of an ultrasound scan), technique planned (with bladder dissection/without bladder dissection), and intention to commence patient on progesterone (yes/no). A “random element” will be included in the algorithm, so that each participant has a probability (unspecified here) of being randomised to the opposite treatment that they would have otherwise received.

#### Concealment mechanism {16b}

Randomisation will be provided by a secure 24/7 online central randomisation system and a back-up free telephone randomisation service (available during working hours).

#### Implementation {16c}

A minimisation algorithm will be used to ensure balance in the treatment allocation by site, indication for cerclage (a history of ≥3 previous midterm losses or premature births (≤28 weeks)/insertion of a cervical suture in a previous pregnancy/a history of mid trimester loss or premature birth with a (current) shortened cervix (≤25mm)/women whom clinicians deem to be at risk of preterm birth either by history or the results of an ultrasound scan), technique planned (with bladder dissection/without bladder dissection), and intention to commence patient on progesterone (yes/no). A “random element” will be included in the algorithm, so that each participant has a probability (unspecified here) of being randomised to the opposite treatment that they would have otherwise received.

### Assignment of interventions: blinding

#### Who will be blinded {17a}

Clinicians cannot be blinded to the allocation. Should suture allocation need to be recorded in the hospital notes to facilitate the placement of the correct randomised suture (e.g. as cerclage is to be performed later by a different clinician), this is acceptable. The suture material used should also be documented within the operation notes. As far as possible, it is preferable that the patient and microbiologists should remain unaware of the treatment allocation. Duration of blinding should continue, where possible, until both mother and baby have reached their respective trial end points (i.e. final follow-up). The primary outcome (miscarriage, stillbirth, termination of pregnancy and neonatal death) is objective and thus should minimise any bias incurred by lack of blinding.

#### Procedure for unblinding if needed {17b}

The suture material used for the cervical cerclage should be recorded on the operation note and this can be accessed. The surgeon cannot be blinded so unblinding will not occur.

## Data collection and management

### Plans for assessment and collection of outcomes {18a}

Data will be collected from the routine medical notes. The sponsor performs independent on site monitoring of patient records to ensure data quality. This is in accordance with a detailed monitoring plan that is available on request with approximately 10% of the clinical records reviewed in detail.

### Plans to promote participant retention and complete follow-up {18b}

The trials team ensures that all outstanding data is queried monthly with all sites. Where babies are not delivered at the site in which the cerclage was performed, follow-up is facilitated through a continuing care site setup processes.

### Data management {19}

Personal and sensitive data will be collected directly from trial participants’ hospital notes. Participants will be informed about the transfer of this information to the C-STICH Study Office at BCTU and asked for their consent. With the patient’s consent, their name, date of birth, National Health Service (NHS) or Community Health Index (CHI) number of both mother and baby, and Hospital number will be securely stored on the trial database. This will enable tracing of women who deliver in a different hospital.

Patients will be identified using only their unique trial number to verify identity on the data collection forms and in any correspondence between the C-STICH Study Office and the participating site.

Consent forms will be collected by the C-STICH Study Office and stored securely in the Trial Master File (TMF). These forms will be available to various regulatory bodies for inspection upon request.

Data collected will be entered onto a secure computer database, either directly by the local site *via* the internet using secure socket layer (SSL) encryption technology, or indirectly from paper forms by C-STICH study office staff. Regardless of whether completing paper or electronic CRFs, only site staff designated the responsibility on the delegation log can do so—in instances where paper CRFs are completed, site staff must provide their name and signature, which BCTU staff will check against delegation log records at the point of receipt. Where data entry is conducted by BCTU staff, data entry quality checks will occur according to predetermined parameters laid out in the Data Management Plan. In addition, a Data Validation Plan will guide BCTU staff in the generation of queries where, for instance, data provided by sites may not fall into any typically expected parameters. The ability to enter or edit site data on the database will only be provided to those site individuals designated the responsibility by their PI on the delegation log. These individuals will receive usernames and passwords and will only have the ability to edit data relating to their site(s). Similarly relevant BCTU staff will also be provided with usernames and passwords but will have the ability to enter/edit data on behalf of all sites, though may only do so in accordance with the Data Management Guidelines. The database will maintain an audit trail of all data entered by any username. BCTU will also adopt a centralised approach to monitoring data quality and compliance. The trial database has been constructed specifically for the trial data and will include range and logic checks to prevent erroneous data entry. Independent checking of data entry will be periodically undertaken on small sub-samples (i.e. data entry quality checks as mentioned above). The trial statistician will regularly check the balance of allocations by the stratification variables.

Trial data will be held on secure BCTU servers, which are backed up periodically.

All staff involved in the C-STICH study, be they clinical, academic, or employees of BCTU, share the same duty of care to prevent unauthorised disclosure of personal information. No data that could be used to identify an individual will be published. Personal data recorded on all documents will be regarded as strictly confidential and will be handled and stored in accordance with the Data Protection Act 1998 and any amendments.

### Confidentiality {27}

All data will be stored and processed under the provisions of the General Data Protection Regulation 2018. Electronic data will be stored on a secure server at Birmingham Clinical Trials Unit (BCTU). Access to participant data is restricted to designated staff at the C-STICH Trial Office and clinicians treating the participants all whom have unique usernames and passwords. Access to the trials unit is by authorised individuals only. All data received in paper format for the trial will be kept in a lockable unit in a secure storage area with access only by C-STICH trial staff.

Published results will not contain any personal data that could allow identification of individual participants.

All investigators and study site staff involved with this study may not disclose or use for any purpose other than performance of the study, any data, record, or other unpublished, confidential information disclosed to those individuals for the purpose of the study. Prior written agreement from the sponsor or its designee must be obtained for the disclosure of any said confidential information to other parties. No data that could be used to identify an individual will be published.

### Plans for collection, laboratory evaluation, and storage of biological specimens for genetic or molecular analysis in this trial/future use {33}

Not applicable. The optional microbiological samples collected in the trial form part of routine care with the result being reported. No samples have been stored for use in the trial or for future use.

## Statistical methods

### Statistical methods for primary and secondary outcomes {20a}

#### Statistical analysis

A separate statistical analysis plan for the quantitative analysis of the C-STICH study will provide a detailed description of the planned statistical analyses. A brief outline is given below. All analyses will be by intention to treat. Every attempt will be made to collect pregnancy outcome data on all women and it is anticipated that missing data will be minimal. Women with missing primary outcome data will not be included in the first instance.

#### Primary analysis

Pregnancy loss will be summarised by treatment arm using frequencies and percentages. A log-binomial model will be used to generate relative risks (and 95% confidence intervals (CI)), adjusting for the minimisation variables. Adjusted risk differences will also be presented (and 95% confidence intervals).

### Interim analyses {21b}

An interim report including the analysis of major endpoints will be provided in strict confidence to a DMEC at intervals of at least 12 months, or to a timetable agreed by the DMEC prior to study commencement. Criteria for stopping or modifying the study based will be ratified by the DMEC. Final analysis will be performed once all women and their babies have reached the end of the follow-up period.

### Methods for additional analyses (e.g. subgroup analyses) {20b}

#### Secondary analysis

Secondary outcomes which are binary will be analysed as per the primary outcome. Continuous outcomes which are deemed to be normally distributed will be summarised using means and standard deviations and a linear model will be fitted to generate adjusted mean differences (and 95% CIs). Continuous outcomes which are not deemed to be normally distributed will be summarised using medians and interquartile ranges and unadjusted differences in medians will be produced with 95% CIs. Time to event data will be summarised using medians and interquartile ranges. A cox regression model will be fitted to generate adjusted hazard ratios (and 95% CIs) and a Kaplan-Meier plot will be produced to assess the data visually. All analyses will be adjusted for the minimisation variables (where possible).

### Methods in analysis to handle protocol non-adherence and any statistical methods to handle missing data {20c}

#### Sub-group analyses and missing data

All sensitivity and subgroup analyses will be limited to the primary outcome only. Subgroup analyses will be limited to indication for cerclage (a history of ≥3 previous midterm losses or premature births (≤28 weeks)/insertion of a cervical suture in a previous pregnancy/a history of mid trimester loss or premature birth with a (current) shortened cervix (≤25mm)/women whom clinicians deem to be at risk of preterm birth either by history or the results of an ultrasound scan), technique planned (with bladder dissection/without bladder dissection), and intention to commence patient on progesterone (yes/no). Sensitivity analyses will consist of an analysis to assess the impact of missing data and an analysis to assess the impact of adherence to the randomisation allocation. This will include an as treated and per-protocol analysis.

### Plans to give access to the full protocol, participant-level data, and statistical code {31c}

Requests for data generated during this study will be considered by BCTU. Data will typically be available within 6 months after the primary publication.

Only scientifically sound proposals from appropriately qualified Research Groups will be considered for data sharing. The request will be reviewed by the BCTU Data Sharing Committee in discussion with the Chief Investigator and where appropriate (or in absence of the Chief Investigator) any of the following: the Trial Sponsor, the relevant Trial Management Group (TMG), and independent Trial Steering Committee (TSC).

A formal Data Sharing Agreement (DSA) may be required between respective organisations once release of the data is approved and before data can be released. Data will be fully de-identified (anonymised) unless the DSA covers transfer of patient identifiable information. Any data transfer will use a secure and encrypted method.

## Oversight and monitoring

### Composition of the coordinating centre and trial steering committee {5d}

#### Trial Steering Committee

The TSC provides independent oversight of the trial, providing advice to the Chief and Co-Investigators and the sponsor on all aspects of the trial and affording protection for patients by ensuring the trial is conducted according to the guidelines of Good Clinical Practice.

The composition of the trial steering committee includes independent members, patient representation and the trial chief investigator.

*Independent Members:*

Professor Harry Gee—Emeritus Obstetrician, University of Warwick

Mr Andrew Elders—Statistician, Glasgow Caledonian University

Dr Ruth Curry—Obstetrician, John Radcliffe Hospital, Oxford

Independent PPI Representative

Ms Joanne Deery

Ms Sarah Machin

*Non-independent Members:*

Mr Philip Toozs-Hobson (Chief Investigator)

Professor Jane Daniels

### Composition of the data monitoring committee, its role, and reporting structure {21a}

#### Data Monitoring and Ethics Committee

##### Determining when clear answers have emerged

If one treatment really is substantially better or worse than any other with respect to the primary outcome, then this may become apparent before the target recruitment has been reached. Alternatively, new evidence might emerge from other sources that any one treatment is definitely more, or less, effective than any other. To protect against this, during the main period of recruitment to the study, interim analyses of the primary outcome and adverse events will be supplied, in strict confidence, to an independent DMEC along with updates on results of other related studies, and any other analyses that the DMEC may request. The DMEC will advise the chair of the TSC if, in their view, any of the randomised comparisons in the trial have provided both (a) “proof beyond reasonable doubt” that for all, or for some, types of patient one particular treatment is definitely indicated or definitely contraindicated in terms of a net difference in the major endpoints and (b) evidence that might reasonably be expected to influence the patient management of many clinicians who are already aware of the other main trial results. The TSC can then decide whether to close or modify any part of the trial. Unless this happens, however, the TMG, TSC, the investigators, and all the central administrative staff (except the statisticians who supply the confidential analyses) will remain unaware of the interim results.

The BCTU Trial office will forward open DMEC meeting minutes to the Sponsor and Funding Body.

Appropriate criteria of proof beyond reasonable doubt cannot be specified precisely, but a difference of at least *p*<0.001 (similar to a Haybittle-Peto stopping boundary) in an interim analysis of a major endpoint may be needed to justify halting, or modifying, the study prematurely. If this criterion were to be adopted, it would have the practical advantage that the exact number of interim analyses would be of little importance, so no fixed schedule is proposed.

The DMEC consists of 3 members independent from the BCTU and sponsor.

Professor Marian Knight (Chair) National Perinatal Epidemiology Unit (NPEU)

Mr Richard Smith—(Consultant Obstetrician) Norfolk & Norwich University Hospital

Mr Graeme MacLennan (Senior Statistician—University of Aberdeen)

### Adverse event reporting and harms {22}

The collection and reporting of adverse events (AEs) will be in accordance with the Health Research Authority’s UK Policy Framework for Health and Social Care. There may be unexpected serious adverse reactions associated with monofilament or braided sutures when used in cervical cerclage. Monofilament or braided sutures have been used to treat cervical cerclage for many years and there is no reason to believe there are adverse biochemical reactions intrinsic to the material of the suture thread, but there may be adverse events arising from the biomechanical properties of the thread. There are also known adverse events of cerclage irrespective of suture material used. It is the responsibility of investigators to notify serious adverse events to the C-STICH Trial Office, who will forward all SAEs to the Chief Investigator. It is the remit of the sponsor or their representative to expedite to the ethics committee within 15 days any serious adverse events (SAEs) that are considered related and unexpected. Where SAEs are expedited to the Research Ethics Committee (REC), the PI will be informed by the C-STICH study team of the RECs response and any actions to be taken.

#### Definitions and reporting requirements adverse events (AE)

An AE is:

- Any unintentional, unfavourable clinical sign or symptom such as:
- Any new illness or infection or the deterioration of existing disease or illness
- Any clinically relevant deterioration in any laboratory assessments or clinical tests, for example continued shortening of the cervix or dilatation.

Non-serious AEs do NOT need to be reported to the Trials Office. A SAE is an untoward event which:

- Results in maternal death*
- Immediately threatens the life of participant**
- Results in hospitalisation or prolongation of existing hospitalisation
- Results in a persistent or significant disability/incapacity
- Is an important medical event that may jeopardise the patient or requires medical intervention to prevent one of the outcomes above.

*All maternal deaths will be reported to BCTU on the SAE Form irrespective of whether the death is related to pregnancy, the cerclage procedure, or an unrelated event. If a participant dies, any post-mortem findings must be provided to BCTU. BCTU will report all deaths to the DMEC, CI and sponsor for continuous safety review. All SAEs deemed to be related and unexpected will be expedited to the REC. **Life-threatening in the definition of a SAE refers to an event in which the mother was at risk of death at the time of the event. It does not refer to an event which hypothetically might have caused death if it were more severe. Important AEs that are not immediately life-threatening or do not result in death or hospitalisation, but may jeopardise the pregnancy or may require intervention to prevent one of the other outcomes listed in the definition above, should also be considered serious. All SAEs for the participant and their baby need to be reported to the Trials Office (the only exception being those SAEs listed as exempt from reporting). Assuming they meet the definition of serious, examples of reportable SAEs (which should be reported on the SAE form) are, but not limited to, the following:

- Premature rupture of membranes within 48 h of the cerclage procedure.
- Infection of the amniotic sac (chorioamnionitis) requiring intravenous antibiotics.
- A miscarriage, preterm delivery, or neonatal death within 48 h of cerclage procedure
- Cervical lacerations at time of cerclage procedure
- Bladder injury as a result of the cerclage procedure
- Retained suture material at cerclage removal
- Congenital malformations, abnormalities identified in the neonatal period
- Postpartum haemorrhage at or greater than 1500ml
- Antepartum haemorrhage that requires a cerclage removal.

There are some SAEs associated with the trial that are already well characterised, where it is highly unlikely that the trial will reveal any new safety information relating to the intervention. Recording of these SAEs will not affect the safety of participants or the aims of the trial. As such, the following SAEs are exempt from reporting to the Trials Office:

- Admission for routine monitoring
- Removal of cervical cerclage more than 48 h after the cerclage procedure
- Treatment, which was elective or pre-planned, for a pre-existing condition that is unrelated to the pregnancy
- Admission to a hospital for delivery of the baby
- Management of a premature baby
- Admission for observation in cases of threatened preterm labour
- Postpartum haemorrhage less than 1500ml
- Antepartum haemorrhage not requiring cerclage removal
- Premature rupture of membranes after 48 h of cerclage procedure
- Preterm labour after 48 h of cerclage procedure
- Miscarriage after 48 h of cerclage procedure
- Congenital malformations, abnormalities identified on the mid trimester scan
- PV bleed
- Anaesthetic complications

A miscarriage, preterm delivery, or neonatal death 48 h after the cerclage procedure will be considered an outcome and not a SAE and should be reported on the pregnancy outcome form. If a site reports an SAE that the Trials Office believes exempt from reporting, clarification will be sought by the Trials Office, with the site asked to review their reasons for reporting. If both parties then agree the SAE is exempt from reporting, the SAE will no longer require processing, though a record of it and the relating correspondence should be kept by both parties. AEs should still be recorded locally in accordance with local practice/SOPs. Reportable SAEs, for both the participant and their baby, from the day of randomisation into the trial, until the defined end of the trial, should be reported to the Trials Office. Whether observed directly or reported by the patient, these must be recorded on CRF 7 within 24 h of the research staff becoming aware of the event. The PI (or nominated investigator (medically qualified doctor) delegated the relevant responsibility on the delegation log) is required to assign causality, signing the SAE form to confirm their assessment. Where an SAE form has been completed by someone other than an investigator, the original SAE form will be required to be countersigned by the Investigator to confirm agreement with the causality. Assessment of causality must be made by an investigator (definition above) according to the definitions in the table below. If an investigator is unavailable, initial reports without causality assessment should be submitted to BCTU by a healthcare professional within 24 h, but must be followed up by medical assessment as soon as possible thereafter, ideally within the following 24 h. If information is missing from the initial SAE report, including ongoing events where there is as yet no end date, the SAE should be followed up until resolution or stabilisation of the event. Follow-up information should ideally be provided on a new SAE Form, using the SAE reference number provided by the BCTU trials team. For all reportable SAEs, the CI or delegate will review the SAE and provide their own assessment of causality. In cases where either the reporting investigator or the CI believes the SAE related the CI or delegate will also perform an expectedness assessment. This expectedness assessment will be based on the clinical judgement/experience of the CI or delegate, with reference where appropriate, to those SAEs listed as expected below. For expectedness assessment purposes, the following will be considered as expected SAEs:

- Preterm prelabour rupture of membranes, miscarriage, neonatal death within 48 h of cervical cerclage procedure
- Antepartum haemorrhage requiring suture removal
- Postpartum haemorrhage greater than 1500ml
- Retained suture thread at cerclage removal
- Postnatal readmission with infection/sepsis

Where there is a discrepancy between the local assessment of causality and the assessment from the CI, neither will be downgraded and both opinions will be documented. If either party believes an SAE related, and the CI believes it unexpected, the SAE will be reported to the REC within 15 days. Details of this will be provided to the relevant site’s PI and should be filed in the relevant section of the Investigator Site File. If an AE is mistakenly reported as an SAE, the site will be informed and the SAE will be downgraded to an AE.

**Table:** 

Category	Definition	Causality
**Definitely related to fitted suture thread**	There is clear evidence to suggest a causal relationship, and other possible contributing factors can be ruled out	Related
**Probably related to fitted suture thread**	There is evidence to suggest a causal relationship, and the influence of other factors is unlikely	
**Possibly related to fitted suture thread**	There is some evidence to suggest a causal relationship; however, the influence of other factors may have contributed to the event (e.g. the patient’s clinical condition, other concomitant events or medication)	
**Unrelated to fitted suture thread**	There is no evidence of any causal relationship	Unrelated

The DMEC reviews this open, unblinded data for safety. BCTU also reports a summary of all SAEs to the TSC, blinded to treatment allocation following a timetable agreed by the TSC prior to study commencement.

### Frequency and plans for auditing trial conduct {23}

The study will be subject to onsite and remote monitoring to ensure compliance with GCP. A risk proportionate approach to the initiation, management and monitoring of the study will be adopted and outlined in the study-specific risk assessment. The sponsor will perform regular onsite monitoring for all sites according to BWH SOPs. The study specific risk assessment will be regularly reviewed in light of monitoring for all sites according to BWH SOPs. The study specific risk assessment will be regularly reviewed in light of monitoring findings to ensure risk proportionate levels of monitoring of data as per BCTU SOPs.

#### Direct access to source data

Investigators and their host Trusts will be required to permit trial-related monitoring and audits to take place by the sponsor representative, providing direct access to source data and documents as requested. The trial site may also be subject to audit by the Research and Development Manager of their own Trust, or monitoring by the sponsor, and should do everything requested by the CI in order to prepare and contribute to any inspection or audit or monitoring. Trial participants will be made aware of the possibility of external audit of data they provide in the participant information sheet.

#### Central monitoring throughout the trial

The study will also adopt a centralised approach to monitoring data quality and compliance. A computer database will be constructed specifically for the trial data and will include range and logic checks to prevent erroneous data entry. Independent checking of data entry will be periodically undertaken on small sub-samples. The trial statistician will regularly check the balance of allocations by the stratification variables.

#### Definition of a serious breach

A serious breach is that which is likely to effect to a significant degree:

1. The safety or physical or mental integrity of the participants of the trial; or

2. The scientific value of the trial.

If a potential serious breach is identified by the Chief investigator, Principal Investigator, or BCTU, the C-STICH Trial Office must be notified within 24 h. It is the responsibility of the Chief Investigator to determine whether the incident constitutes a serious breach and if so, to assess the impact of the breach on the scientific value of the trial. BCTU will report serious breaches to the sponsor and to the Research Ethics Committee as necessary.

### Plans for communicating important protocol amendments to relevant parties (e.g. trial participants, ethical committees) {25}

The conduct of the trial will be according to the principles of the International Committee on Harmonisation, Good Clinical Practice Guidelines (ICH GCP). All centres will be required to sign an Investigator’s Agreement, detailing their commitment to accrual, compliance, GCP, confidentiality, and publication. Deviations from the agreement will be monitored and the TSC will decide whether any action needs to be taken, e.g. withdrawal of funding, suspension of centre. The Trial Office will ensure researchers not employed by an NHS organisation hold an NHS honorary contract for that organisation. The Trial has a favourable ethical opinion from Cambridgeshire and Hertfordshire Multi-centre REC, confirming that the trial design respects the rights, safety, and wellbeing of the participants. The Comprehensive Research Network will conduct governance checks and assess the facilities and resources needed to run the trial, in order to give host site permission. For sites in Scotland and Wales, the Trial Office is able to help the local PI in the process of the site specific assessment by completing much of Site Specific Information section of the standard IRAS form as possible. For sites in England, the trial office is able to help with Trust confirmation of capacity and capability by helping to complete the Statement of Activities and Schedule of Events. The local PI will be responsible for liaison with the Trust management with respect to locality issues and obtaining the necessary signatures at their Trust. Once the following has taken place for each Trust, Health Board, or NHS Board, the Trial Office will send a folder containing all trial materials to the local Principal Investigator along with a letter of activation, and potential trial participants can then start to be approached:

- Sites in Scotland have received NHS Board permission for the study
- Sites in Wales have received Health Board approval for the study
- Sites in England have received Trust confirmation of Capacity and Capability
- The contract agreement for the Trust/Health Board/NHS Board is fully executed
- The site initiation visit has taken place
- A delegation log has been completed by the PI and sent to the trial office
- A CV and GCP Certificate for the PI has been sent to the trial office

Within 90 days after the end of the study, the CI will on behalf of the Sponsor ensure that the REC is notified that the study has finished. If the study is terminated prematurely, those reports will be made within 15 days after the end of the study.

The CI will supply the Sponsor with a summary report of the clinical study, which will then be submitted to the REC within 1 year after the end of the study.

## Dissemination plans {31a}

CSTICH is registered on the ISRCTN registry.

A meeting will be held after the end of the study to allow discussion of the main results among the collaborators prior to publication. C-STICH is intended for publication via peer-reviewed scientific journals and via the C-STICH website.

## Discussion

### Trial status

The protocol is currently version 9.0, approved on 25 March 2020.

The trial opened to recruitment in August 2015 and the first recruit was recruited on 22 September 2015. Recruitment completed in January 2021 and the trial is currently in the follow-up phase, with results anticipated in November 2021.

## Abbreviations

AE: Adverse event
BCTU: Birmingham Clinical Trials Unit at the University of Birmingham
CI: Chief Investigator
DMEC: Data Monitoring and Ethics Committee
GCP: Good Clinical Practice
GP: General Practitioner
ISD: Information Services Division
ISRCTN: International Standard Randomised Controlled Trial Number
PI: Principal Investigator—the local lead investigator for the C-STICH Trial
PIS: Participant Information Sheet
REC: Research Ethics Committee
SAE: Serious adverse event
SOP: Standard operating procedure
TMG: Trial Management Group
TSC: Trial Steering Committee
